# GDSC SMLM: Single-molecule localisation microscopy software for ImageJ

**DOI:** 10.12688/wellcomeopenres.18327.1

**Published:** 2022-09-29

**Authors:** Thomas J. Etheridge, Antony M. Carr, Alex D. Herbert

**Affiliations:** 1 Genome Damage and Stability Centre, School of Life Sciences, University of Sussex, Falmer, BN1 9RQ, UK

**Keywords:** SMLM, single-molecule, super-resolution, ImageJ, Java, fluorescence imaging, software

## Abstract

Single-molecule localisation microscopy (SMLM) uses software to extract super-resolved positions from microscope images of fluorescent molecules. These localisations can then be used to render super-resolution images or analysed to extract information about molecular behaviour. The GDSC SMLM software provides a set of tools for analysing SMLM data in a single cross-platform environment. The software identifies fluorescent molecules in raw microscope images and localises their positions using stages of spot detection, spot fitting and spot rejection. The resulting localisation data set can then be visualised, cropped and filtered. A suite of downstream analysis tools enable the user to perform single-particle tracking, cluster analysis and drift correction. In addition, GDSC SMLM also provides utility tools that enable modelling of EM-CCD and sCMOS cameras as well as point spread functions (PSFs) for data simulation. The software is written in Java and runs as a collection of plugins for the ImageJ software.

## Introduction

Single-molecule localisation microscopy (SMLM) uses image processing software to extract super-resolved positions of individual fluorescent molecules from diffraction-limited time series of microscope images ^1– 3^. Depending on the sample type, single-molecule localisation data sets can be used to reconstruct pointillist super-resolution images of cellular structures or extract information about molecular diffusion. A range of SMLM-based techniques now exist, each with differing strategies for temporally separating fluorescence emission from closely spaced fluorescent molecules ^4^. The application of these techniques relies heavily on the availability and usability of SMLM analysis software. Over the past decade, many research groups have sought to develop their own custom software solutions for analysis of single-molecule data to maximise the flexibility and clarity of analyses which is otherwise not achievable with proprietary software. In a similar vein, we aspired to create an all-in-one solution for our data analysis that required no programming experience from the end user and could be easily expanded as new techniques and methodologies emerged. Here, we describe the resulting Genome Damage and Stability Centre (GDSC) SMLM software for single-molecule localisation and analysis, available in a single cross-platform software environment as a set of plugins for I mageJ ^5^ (RRID:SCR_003070).

The GDSC SMLM [40] software encompasses a single-molecule fitting plugin, P eak F it, which can determine the position of fluorescent molecules appearing as spots in raw localisation microscopy image sequences. The performance of this plugin was ranked as one of the best-in-class for the 2D data sets in the second localisation microscopy software challenge ^6^. It uses a hybrid approach to fit spot candidates that combines simultaneous multi-emitter fitting ^7– 9^ and single-emitter fitting ^10^. Data sets of single molecule positions and associated metrics ( *e.g.* localisation precision) can then be visualised in table format or rendered into super-resolution images.

A wide range of supplementary plugins are also available for quantitative analysis of SMLM data. For example, subsets of the localisation data sets can be produced using filters or by cropping using regions of interest on rendered images. Plugins for more in-depth analyses are also provided for techniques such as single-particle tracking, clustering and cluster visualisation ^11– 13^, pair-correlation photoactivated localisation microscopy (PC-PALM) ^14– 17^, time-correlated photoactivated localisation microscopy (tcPALM) ^18^, cross-talk activation analysis ^19^ and Fourier image resolution ^20^. Fitting and analyses capabilities are supported by a suite of calibration and modelling plugins which allow analysis of the noise and gain of electron multiplying charge-coupled device (EM-CCD) and scientific complementary metal–oxide–semiconductor (sCMOS) cameras for use in maximum likelihood fitting models, as well as construction of point spread function (PSF) models from fluorescent bead calibration images. Finally, a group of simulation plugins allow users to create SMLM-like camera images for quantitative testing of models and predictions.

The GDSC SMLM software has been successfully used in recent single-molecule studies to quantify chromatin association of DNA binding proteins in fission yeast ^21– 23^, visualise clustering of glucose receptors in adipocytes ^24^ and calculating single-molecule dwell times of EB3 on microtubules *in vitro* ^25^. In this paper we provide examples of elementary use cases that describe fitting localisation image data, handling localisation data, image rendering and data analysis for single molecule tracking experiments. The software is supported by an online user manual of the available functionalities, providing comprehensive documentation including a workflow for the optimisation of fitting parameters for typical imaging conditions.

## Methods

### Analysis methods

**Figure 1.**
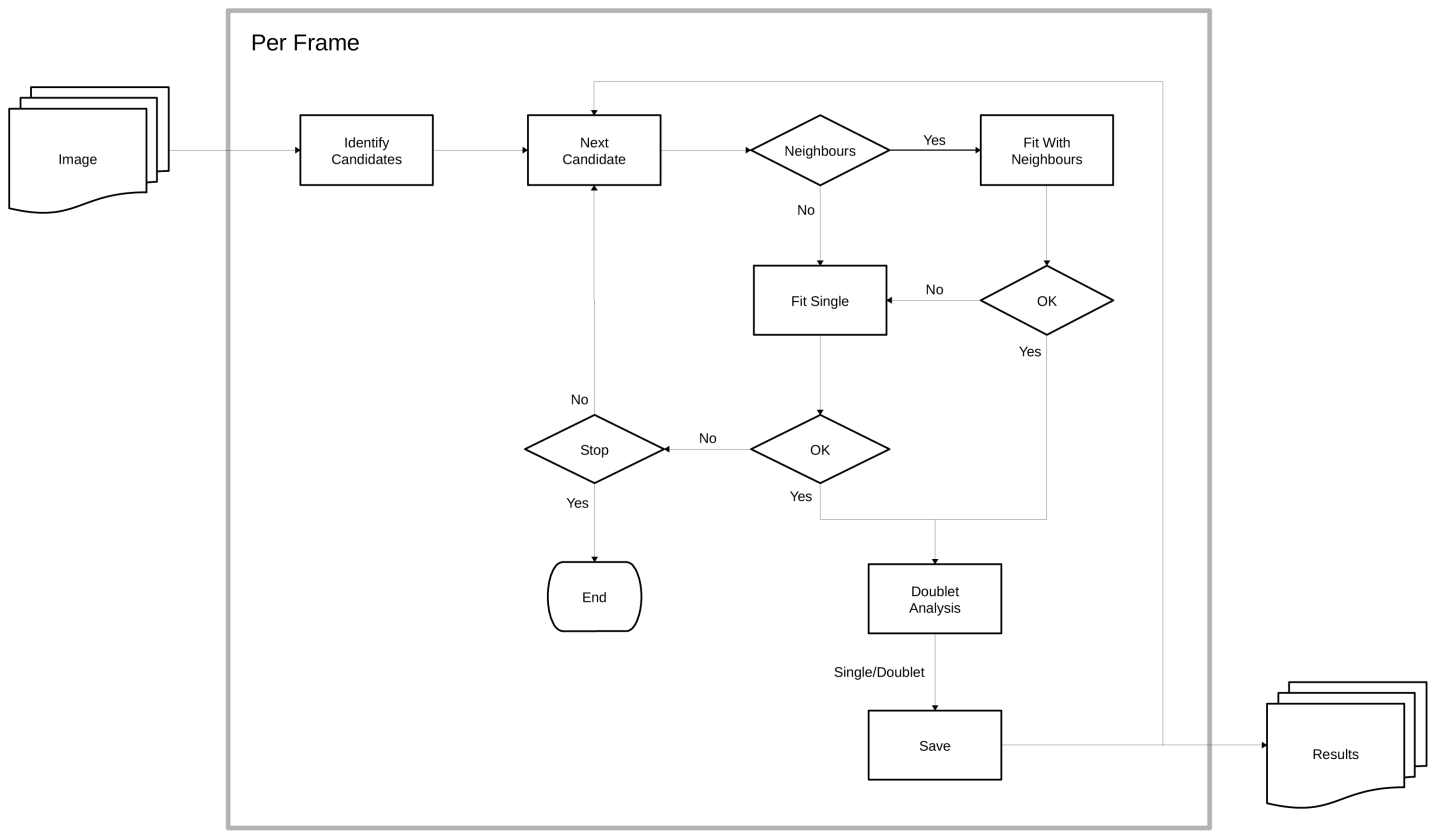
P eak F it image processing pipeline. Image frames are processed independently. Spot candidates are identified, ranked and processed sequentially by fitting a Gaussian 2D point spread function (PSF). Fitted PSFs must pass a filter that rejects fits that deviate from the expected shape and position, or are of low signal or localisation precision. Any neighbours within the fit region are included for a multi-spot fit; failed multi-fits revert to single spot fitting. Successful fits are analysed for unaccounted for signal and may refit as two spots (doublets). Fit results are saved for reuse during fitting of neighbours and output to the final results. Failed fits may signal the end of frame processing based on stopping criteria.

Single molecule image data consists of single point sources of light which are then subjected to the point spread function (PSF) of the microscope. The P eak F it plugin uses a 2D Gaussian function to model the PSF and is suitable for PSFs that appear as spots on the image. Figure 1 shows an overview of the image processing pipeline. Fitting the image data involves identification of candidate spots; fitting the spots using the PSF; and filtering the results to reject poor fits. Image frames are processed independently allowing parallel processing.

The identification stage finds spot candidates within a box region using non-maximum suppression ^26^. Typically the region is a square of edge length 2 *n* + 1 where *n* = ⌊ *Aσ*_0_⌋, *σ*_0_ is the initial Gaussian width and *A* is the search parameter. Noise can be reduced using a smoothing filter prior to identification, for example a mean or Gaussian filter.

Fitting uses a Gaussian function as described in Smith *et al.*, 2010 ^27^ to model the signal for each pixel as


$$u_{k}(x,y)=B+Signal\times \text{Δ}E_{x}(x,y)\times \text{Δ}E_{y}(x,y)\;(1)$$

with: *x* and *y* the centre of the k ^th^ pixel; *u _k_* ( *x*, *y*) the expected value in the k ^th^ pixel; *B* the background level; *Signal* the total volume of the Gaussian; ∆ *E _x_* ( *x*, *y*) the integral of the Gaussian 2D function over the x-dimension; and ∆ *E _y_* ( *x*, *y*) the integral of the Gaussian 2D function over the y-dimension.

The fitting stage is a single pass algorithm which visits each candidate only once. The spot candidates are ranked by intensity and processed in order during fitting. Fitting uses a box region around the candidate, typically the region is a square of edge length 2 *n*+1 where *n* = ⌊ *Bσ*_0_⌋ and *B* is the fitting parameter. For each candidate the algorithm selects from several possible fitting options depending on whether other candidates are within and/or adjacent to the fitting region. The target candidate is always fit and the XY position is allowed to freely move.

In high density single-molecule data, it is possible for multiple emitters to be present in the fit region - these candidates are known as neighbours. These are included in the fit if their intensity is within a fraction of the intensity of the target; typically the neighbour height is 30%. Neighbour candidates have their XY position bounded by a shift of *±*1 pixel. Any candidates that have previously been visited use their known fit parameters to initialise fitting; unprocessed candidates use an estimation routine to initialise the fit parameters using the peak height and expected PSF width. The fit region is expanded by 50% to define an area of pixels outside the region. If these contain previously fitted spots the PSF of each spot is subtracted from the data (these are precomputed neighbours) to remove bright pixels at the border of the fit region. If fitting using multiple PSFs fails any previously fitted neighbours have their PSF subtracted from the data before a second fit of only the target spot. In the event of low density data with no neighbour candidates the algorithm defaults to fitting only the target candidate. The Levenberg-Marquardt algorithm (LMA ^28, 29^) is used to fit the PSF. A camera calibration allows converting the input data to photons for fitting using maximum likelihood estimation (MLE) for Poisson distributed data ^30^. If calibration is not available then fitting uses a least-squares estimator (LSE).

If the fitting successfully converges the target spot may be refit as a pair of spots (doublet). This is only performed if the fit residuals (the difference between fitted function and the actual data) are asymmetric. Asymmetry analysis is performed using an adaption of the method detailed in the rapi *d*STORM ^10^ user documentation and is redescribed here for clarity. The residuals are divided into four quadrants surrounding the fit centre labelled clockwise *A* to *D*. Opposing quadrants are summed and the absolute difference divided by the total sum


$$\text{Score}=\frac{\left|(\sum A+\sum C)-(\sum B+\sum D)\right|}{\sum \left|A\right|+\sum \left|B\right|+\sum |C|+\sum \left|D\right|}\;(2)$$

Analysis is performed using axes XY centred on the fit location to define the quadrants, and repeated with the axes rotated 45 degrees. The candidate is refit as two spots if the maximum residuals score is above a threshold. The pair of spots are accepted if they pass the configured spot filter and the fit score is improved. Improvement is measured for least squares estimation using the adjusted coefficient of determination ^31^; maximum likelihood estimation methods use the Bayesian Information Criterion ^32^.

The fitting stage can perform up to four fits per target: candidate fit with neighbours (multi); candidate fit as doublet with neighbours (multi doublet); candidate fit (single); and candidate fit as doublet (single doublet). Fits are performed as required. If the fit with neighbours is accepted then the single fit is not performed. If there are no neighbours or the neighbour fit failed then the single fit is performed. Success for either the multi or single fit may trigger a doublet fit depending on the residuals analysis.

The filtering stage uses various filters based on the fit parameters to reject or accept the spot. The initial standard deviation *σ*_0_ is compared to the fitted standard deviation and assessed using a minimum and maximum width factor. The initial target position is compared to the fitted position and assessed using a shift factor. The signal-to-noise ratio (SNR) is computed using the mean signal of the Gaussian within the region defined by the peak width at half maxima (PWHM), and the noise estimated from the fitted background assuming a Poisson noise model with added Gaussian read noise of the camera


$$\;\bar{I}=\frac{I}{2\pi \sigma_{x}\sigma_{y}r^{2}}\;(3)$$

$$\;\text{SNR}=\frac{\bar{I}}{\sqrt{B+\frac{\sum^{n}var_{i}/g_{i}^{2}}{n}}}\;(4)$$

with: $\bar{I}$ the mean Gaussian intensity; *I* the Gaussian intensity in photons; *σ* the X or Y standard deviation; *r* the Mahalanobis distance for a 2D normal distribution that contains 50 percent of the integral ( *r* = $\sqrt{-2\ln(1-p)}$ ; *p* = 0.5); *πσ _x_σ _y_ r* ^2^ the area of the Gaussian containing half the signal; *B* the local background in photons computed in a local background region; *var _i_* the variance at pixel *i* in counts; *g _i_* the gain at pixel *i*; and *n* the number of pixels in the background region. The background region size is 2 *w* + 1 defined as *w* = ⌈ *rσ*⌉ clipped to [1, 3] in each dimension. For single spots the local background is the fitted background plus the contribution to the local region from precomputed neighbours. For multi spots the background is the mean of the input data in the local background region with the candidate spot subtracted. The SNR must pass a minimum threshold. The XY localisation precision is computed using the Mortensen formula ^33^, or derived from inversion of the Fisher information matrix for a Poisson process (see Smith *et al.*, 2010, SI eq. 9 ^27^). The individual filters are combined to create a composite selection criteria used to accept the spot parameters. 

Candidates are processed per frame in order of intensity and processing is halted based on stopping criteria. The fail limit specifies the number of consecutive failures that are allowed before stopping. The pass rate specifies the fraction of fits that must be successful otherwise processing is stopped. If the stopping criteria is reached no further unvisited candidates will be fit. However any low ranking candidates that were fit as neighbours will be processed as the main fitting target to refine the parameters generated when the fitting region was not centred on the spot.

### Implementation

The GDSC SMLM software (RRID:SCR_022717) is written in Java and structured into two components: GDSC SMLM contains all the code for single molecule analysis; and GDSC Core [41] contains general utilities and is used by software other than the SMLM code ^34^. Each component is divided into two modules: a base module contains the analysis functionality and can be used directly as a library; and a module that requires the I mageJ ^5^ library as a dependency and is intended to be executed by I mageJ in a graphical environment. The GDSC SMLM I mageJ module contains plugins that function to collect input parameters, execute the library routines and present results.

The software uses a data model of localisation microscopy results. The model contains the XYZ coordinates of each molecule and the associated data generated when processing raw image data such as signal intensity, noise and localisation precision. The model also contains metadata describing the microscope used in the data acquisition such as the image bounds, pixel magnification, camera specification and PSF information. The calibration is used to map the raw image data such as pixel position and camera counts to physical units such as position in nm and intensity in photons. The data model provides an application programming interface (API) to access data in specified units allowing storage-agnostic data analysis.

### Operation

The GDSC SMLM software requires Java 1.8. There are no platform requirements beyond those required to run I mageJ and the software has been tested on Windows, Linux and Mac OS. The software is packaged into Java archives (jars) for the GDSC Core and GDSC SMLM components. There are a number of dependencies that are required at runtime. The software is distributed using an I mageJ update site which hosts all the required files to install the software into an instance of I mageJ. For example a user of Fiji ^35^ (RRID:SCR_002285) should run H elp > U pdate and add the GDSC SMLM2 update site. This will install and regularly update the software to the latest version. The software can be installed manually by downloading the latest jar files from the update site here and placing them in the I mageJ plugins and jars directories. Install instructions are available in the online manual available here.

The plugins are under the P lugins > GDSC SMLM menu and grouped by general functionality (see Table 1). A tools window can be opened that provides buttons to execute each of the plugins. This can be customised to change the order and available plugins by editing a configuration text file to allow grouping common plugins. The plugins have been designed to support the I mageJ macro recorder and batch execution in macros. Settings are collected using dialogs and a Help button will open a web page for the user manual describing all the parameters for the plugin. Dialogs may collect additional options for currently configured settings using context sensitive buttons.

**Table 1.** Available sub-menus of I mageJ plugins under the P lugins > GDSC SMLM (Genome Damage and Stability Centre single-molecule localisation microscopy) menu.

Sub-menu	Description
Fitting	Identification of localisations on an image.
Results	Loading, saving and management of localisation results sets.
Analysis	Analysis on localisations for example single particle tracking, clustering and Fourier image resolution.
PC PALM	Pair correlation (PC) analysis.
Model	Simulate single-molecule images.
Calibration	Estimate point spread function widths and allow calibration of the imaging camera noise and gain.
Tools	Utility plugins for image manipulation.
Toolset	Install of the SMLM Toolset and configuration of the SMLM Tools window.

Analysis is performed on images or previously generated localisation data sets. The P eak F it plugin is used to fit a 2D Gaussian PSF to single molecule imaging data. The plugin can be executed on the current image or against an image series in a specified folder. Precomputed results can be loaded from file. Custom file formats can be loaded using the L oad L ocalisations plugin which reads any delimited text file using a configurable text parser. The R esults M anager is used to load files to memory, display results and save analysis results to file. The software provides text and binary file formats supporting all localisation data and metadata. The analysis plugins operate on localisation data, without assumptions on the original image PSF, and may create graphic output, files or new data sets. Data sets may be exported in various formats for analysis in external software.

## Use cases

The following use cases provide an introduction to functionality in the GDSC SMLM software. The sections detail fitting single molecule localisation data; and loading, displaying, analysing and saving localisation datasets. A data set containing example use case data is available here ^36^. The data set provides an SMLM image, a fit settings template, and the results of fitting the image using the template settings (see the *Data availability* section for details).

### Fitting single molecule localisation data

Fitting single molecule localisation data requires a series of input image frames. This can be a stack image open in I mageJ or a file series loaded from a folder. OME TIFF images too large to fit in memory can be opened using the TIFF S eries V iewer plugin from the GDSC SMLM T ools menu.

The image was opened in I mageJ and the P eak F it plugin was run. The dialog contains settings for calibration of the input image, spot filtering, spot fitting, fit result filtering, results output and results preview. The preview option allows the results to be displayed for the current frame, and allows interactively changing the settings and the image frame.

Calibration of the input image pixel size and exposure time is required to generate results in physical units. Details of the camera used to capture the image is required for maximum likelihood fitting. If the camera type is unknown then fitting is limited to least-squares estimation. CCD cameras require the camera bias, gain and read noise. sCMOS cameras require a camera model containing per-pixel calibration; a model can be generated from calibration images using the SCMOS A nalysis plugin.

The localisations are fit using a Gaussian 2D function to model the PSF. The type of function can be selected and the PSF width parameters provided. The width can be estimated from observations on fixed fluorophores imaged at various z-depths. The value should represent the width of the in-focus PSF, *i.e.* the minimum of the width against z-depth profile. An approximate value, typically around 1 pixel, can be used to generate results and the average width of high quality spots used to refine the PSF width.

The spot filtering settings control identification of candidate spots. A wider smoothing filter will reduce the number of candidates by eliminating noise but may also merge close neighbours to a single candidate; a wider search width will reduce the number of candidates in noisy regions but may eliminate neighbours in dense regions; the border width prevents fitting of candidates near the edge of the image; and the fitting width controls the extent of the fit window around the spot. A wider fitting width will improve accuracy for isolated spots at the expense of speed however high density regions may be very slow if neighbours are included in the fit. Ideally the width should cover most of the PSF through the entire depth of field where spots appear as Gaussian peaks. The effect of changing the spot filter parameters can be explored dynamically on an image using the S pot F inder (P review) plugin. This uses the same spot filter configuration and previews the candidates on the image.

The fitting settings specify the fit solver and the fit engine configuration. The fit solver chooses the method used to fit the data. Least-squares fitting can be used without any camera calibration. The other methods require the camera information to create the probability model for fitting. There are several maximum likelihood estimation (MLE) methods available; the authors find the Levenberg–Marquardt method for Poisson distributed data ^30^ is suitable for most images as a compromise between speed and robustness. Further details of the fit solvers and their suitability for different data can be found in the user documentation. Fit solver configuration is collected using an additional options dialog including parameters controlling the convergence criteria. Increasing the number of iterations can be used to improve the number of fits that converge. High density data benefits from higher iterations at the expense of speed. Details of the parameters for each fit solver are in the user manual accessed from the Help button. The fail limit specifies how many candidates are allowed to be rejected before stopping fitting of the image frame. Processing also stops when the fraction of successful candidates is below the pass rate. Neighbours can optionally be included in the fit if they are above a height threshold relative to the candidate. A value too low can include candidates that are image noise. Low neighbours typically do not affect the fit of a peak whereas higher neighbours contain most of the signal in the fit region. The residuals threshold is for high density data. It controls how asymmetric a spot must be to refit as two spots; any spot with residuals above this threshold is refit as a doublet. Doublet fits are only accepted if they pass the results filter and the fit score is an improvement over the single fit. Lowering the neighbour height and residuals threshold impacts runtime and these parameters can be adjusted by repeat fitting of data and monitoring runtime and fitting performance. The duplicate distance is used to exclude any fit result close to an existing result in the frame to eliminate drift from a candidate location to another spot in the fit region. 

The result filter settings control selection of the fit results. Results must pass the configured criteria using measures such as how far the fit shifted from the candidate location, the signal-to-noise ratio (SNR), the fitted width compared to the initial width, and the estimated localisation precision. A simple filter rejects the fit result if any of the configured criteria are not satisfied. Alternatively it is also possible to specify a smart filter that supports logical combinations (And, Or) to create complex filter logic (for details see the user manual). The SNR and precision filters use the signal and width of the fitted Gaussian and the background noise and are the best filters to exclude poor fit results. The minimum width filter can be used to exclude fits that are too narrow to be a PSF and are false positive candidates from image noise. The maximum width filter can be used to limit the depth of field since out of focus spots will have a wider PSF. If fitting diffusing molecules the spot may be wider due to motion blur and the width filter should be configured wider to accommodate the PSF blur.

The results settings control the results output. The Log progress option will output verbose fitting information on each candidate; it can be used to gain information on fitting a specific target on an example frame including why a fit failed or was rejected. This information assists in setting the parameters. Results may be output to a table, rendered into an image, and saved to file or memory. If no output options are selected the default is saved to memory. In-memory results can be output to the other formats using the R esults M anager plugin.

When the settings are configured the OK button will start fitting on the image. The fitting progress is reported to the I mageJ progress bar and can be stopped using the Escape key. If results were saved to memory the localisations can be viewed on the input image using the O verlay R esults plugin. Renaming the results can be performed using the R ename R esults plugin allowing repeat fitting with different settings to be compared using the R esults M atch C alculator plugin.

### Template settings

Templates provide reusable settings for localisation fitting. Templates can be used to pre-configure settings for the software for different microscope equipment or imaging conditions. A template can be created using the F it C onfiguration plugin. This presents the current settings used in localisation fitting. These can be adjusted if required, including using any current templates as a start point, and then saved as a template file. The template is registered with the software and available for use when fitting an image. The template will be reloaded for the next session in I mageJ.

Templates are managed using the T emplate M anager plugin. This allows the current templates to be viewed, new templates to be registered and existing templates to be deregistered. Templates are divided into two classes: standard templates are built into the software and provide default settings that are suitable for a range of input images; custom templates are stored as files and registered. When viewing a custom template the file path will be shown allowing the template file to be transferred to and registered with another I mageJ instance.

### Loading and saving localisation data

Localisation results can be read from and written to supported formats using the R esults M anager. The GDSC SMLM text file format uses tab delimited fields that can be read by other software. The file contains header information describing the results such as the calibration, coordinate bounds and if applicable the fit configuration used to generate the results. A binary format can be used to support faster I/O (input/output) of large data sets.

Plain text localisation files in any delimited format can be read using the L oad L ocalisations plugin. The field delimiter can be configured and the columns in the data assigned to the required localisation fields of time frame and coordinates. Optional fields such as signal intensity, estimated localisation precision and molecule IDs can be read. When loading a localisation file the calibration can be specified for the distance and intensity units and information on the camera can be provided. This information is used by analysis plugins to interpret the localisation data in meaningful physical units. A data set loaded into memory can have the calibration updated using the C alibrate R esults plugin. Results can be written in a custom delimited text format using the S ave L ocalisations plugin which writes any of the available fields to file in a user-specified format.

### Results display

Localisation data sets can be displayed in a table or rendered into a image using the R esults M anager. The input localisations can be read directly from file or chosen from data sets held in memory. An interactive result table shows the required localisation data of time frame and coordinates and provides a configurable display of optional data such as fitting data and image noise data. Subsets of the displayed data can be created by deleting rows and saved to the same or a new results set in memory. Data can be sorted by a chosen column. If the original image used to generate the data is open in I mageJ the table can overlay the current selection on the image. The table can open the original image if the data set contains the image file information and the file is available.

Images are rendered using a scaling of the localisation coordinates to output pixels. The reconstruction maps each localisation to a pixel and assigns the chosen magnitude to the single pixel or weighted to the 2x2 surrounding neighbours. The magnitude can be assigned as a single count, or using localisation data such as the localisation intensity, frame, z depth or ID. Optionally localisations with PSF information can be rendered using a 2D Gaussian to approximate the spot. Each additional localisation mapped to the same pixel creates an update that is an addition for intensity data or a replacement for non-intensity data such as frame or ID. The histogram equalisation option performs contrast enhancement to improve visibility of low intensity pixels. The final image is created as an I mageJ image.

### Single molecule tracking

Localisations can be joined into continuous tracks representing a molecule’s movement over time. Localisations are assigned an identifier for the track and can be assigned a category identifier within the track, for example to label different diffusion states of the molecule over the track lifetime. Pre-computed tracks can be loaded if the input localisation file contains the track and/or category IDs. This allows existing track data sets to be processed identically to tracks computed within the software in analysis of track lengths, track populations and display of tracks. Tracks can be saved using the GDSC SMLM file format which records the track IDs or exported in formats suitable for analysis by other software such as anaDDA ^37^ and SpotOn ^38^ using the T race E xporter plugin.

Tracking can be performed on existing data sets. A simple tracking algorithm joins localisations if the distance is within the configured time and distance thresholds. Ties are resolved using nearest-neighbour variations which rank with time or distance priority. This is suitable for low density data with short lived tracks. Alternatively, dynamic multiple target tracking ^39^ uses a model to assign the probability that a localisation should connect to a track based on the current diffusion rate and intensity of the molecule. New tracks are created as required and existing tracks can expire if no localisations have been assigned to them for a set number of frames. The algorithm is suitable for long lived tracks as the probability model is constructed using a temporal window of the most recent localisations in the track.

## Conclusions

The GDSC SMLM software provides a wide range of functionality for working with single-molecule localisation microscopy data. Microscope images of fluorescent spots can be processed to super-resolved positions of molecules using the P eak F it plugin. The fitting engine uses the stages of spot identification, localisation and rejection. Each stage is configurable and settings can be saved as templates for repeatable analysis of images from different microscopes and reproducible analysis across software platforms.

Analysis plugins act on localisation data sets that are created by fitting data or loaded from external sources. The I mageJ graphic environment allows data sets to be viewed as images and tables and interactively modified for example by cropping, selecting sub-sets or filtering based on properties of the localisations. Data sets can be saved with all associated data using the GDSC file formats or exported to selected formats for analysis by external tools.

A wide range of plugins are available for analysis such as single-particle tracking, clustering and cluster visualisation, drift correction, tcPALM ^18^, and Fourier image resolution ^20^. Tools are provided for analysis of EM-CCD and sCMOS cameras and construction of PSF models from bead calibration images for use in simulations.

The GDSC SMLM software is distributed as a collection of plugins for I mageJ with a single-click install process using the I mageJ update site. Plugins support recording and playback via the I mageJ macro language and context-sensitive help links to the online documentation. Further details of all the functionality is described in the online user manual.

## Data Availability

Figshare: Single molecule localisation microscopy image using Nse4-HaloTag budding yeast and JFX650 fluorophore. https://doi.org/10.6084/m9.figshare.20795677 ^36^. This project contains the following underlying data: BuddingYeast_Nse4-Halo_10ms.tif. (SMLM image using Nse4-HaloTag budding yeast and JFX650 fluorophore.) BuddingYeast_Nse4-Halo_BrightField.tif. (Brightfield image of the yeast cells.) BuddingYeast_Nse4-Halo_10ms_Fit_Template.txt. (Example template for the GDSC SMLM software to allow fitting the localisations using P eak F it.) BuddingYeast_Nse4-Halo_10ms.tif.results.xls. (GDSC SMLM results file containing the fitted localisations.) BuddingYeast_Nse4-Halo_10ms.tif.exported.csv. (Exported results file containing the fitted localisations.) Data are available under the terms of the Creative Commons Zero "No rights reserved" data waiver (CC0 1.0 Public domain dedication).

## Software availability

1. GDSC SMLM source code available from: https://github.com/aherbert/gdsc-smlm
2. GDSC Core source code available from: https://github.com/aherbert/gdsc-core
3. Archived GDSC SMLM source code available from: https://zenodo.org/record/7107424 ^40^
4. Archived GDSC Core release source code available from: https://zenodo.org/record/7101458 ^41^
5. License: GNU General Public License version 3
