# Supplementary material for: GDSC SMLM: Single-molecule localisation microscopy software for ImageJ
Source: Wellcome Open Res. 2022 Sep 29;7:241. [Version 1] doi: 10.12688/wellcomeopenres.18327.1 (PMC10282564; doi:10.12688/wellcomeopenres.18327.1)
Supplement: Peer review history

# Peer review: Reviewer response for version 1

**DOI:** 10.21956/wellcomeopenres.20314.r58008

**Published:** 2023-06-13

**Authors:** Yiming Li

**Affiliations:** 1 Department of Biomedical Engineering, Southern University of Science and Technology, Shenzhen, Guangdong, China

The authors propose an ImageJ software GDSC SMLM that could analyze SMLM images using a collection of plugins. The software is open-source and well-documented. It offers a range of methods for extracting super-resolution positions, such as identifying single molecule points, PSF fitting, filtering, and visualization. Additionally, it enables quantitative analysis of biological super-resolution images, including single particle tracking, cluster analysis, and drift correction. Followings are some comments about this work:

1. The article and online manual describe that the single-molecule localization method used in the GDSC SMLM plugins is based on 2D Gaussian fitting. It is a pity that the software does not offer 3D PSF fitting functionality. It would be nice if the software could include some more advanced PSF models, such as spline interpolated experimental PSF models, for 3D localization.
2. GDSC SMLM plugins provide a detailed online manual, but I encountered some issues while attempting to run the provided open-source data (https://figshare.com/articles/dataset/Single_molecule_localisation_microscopy_image_using_Nse4-HaloTag_budding_yeast_and_JFX650_fluorophore/20795677). I first installed the latest version of Fiji and updated the GDSC SMLM2 plugins on a workstation following the instructions in the online manual. However, when using the "Simple Fit" plugin to analyze the provided open-source data "BuddingYeast_Nse4-Halo_10ms.tif," I encountered the following error message: "uk.ac.sussex.gdsc.smlm.data.config.ConfigurationException: Invalid number of parameters." Then I reinstalled the GDSC SMLM plugins on a desktop computer, but no configuration file was automatically generated in the ".gdsc.smlm" folder. So, I followed the instructions in the online documentation's Configuration Wizard to configure the camera parameters. However, I am unsure if the camera parameters set according to the online documentation are suitable for the "BuddingYeast_Nse4-Halo_10ms.tif" data because there is a significant difference between the results obtained using the "Simple Fit" plugin and the directly provided "BuddingYeast_Nse4-Halo_10ms.tif.exported.csv" file. I suggest providing a quick user manual step-by-step for the provided open-source data.
3. It would be helpful to include experimental super-resolution images to demonstrate its effectiveness. Additionally, it is recommended to provide a comparison with similar software.

Are the conclusions about the tool and its performance adequately supported by the findings presented in the article?

Yes

Is the rationale for developing the new software tool clearly explained?

Yes

Is the description of the software tool technically sound?

Yes

Are sufficient details of the code, methods and analysis (if applicable) provided to allow replication of the software development and its use by others?

Yes

Is sufficient information provided to allow interpretation of the expected output datasets and any results generated using the tool?

Yes

Reviewer Expertise:

Super Resolution Microscopy

I confirm that I have read this submission and believe that I have an appropriate level of expertise to confirm that it is of an acceptable scientific standard.

# Peer review: Reviewer response for version 1

**DOI:** 10.21956/wellcomeopenres.20314.r58007

**Published:** 2023-06-06

**Authors:** Johannes Hohlbein

**Affiliations:** 1 Wageningen University & Research, Wageningen, Gelderland, The Netherlands

The manuscript “GDSC SMLM: Single-molecule localisation microscopy software for ImageJ” by Etheridge et al. introduces an extensive collection of tools to computationally analyse experimental data of recorded single fluorescent emitters. Many different software packages exist for various frameworks (Python, Java, Matlab, etc) that all differ greatly in their functionality and accessibility. In the Java world (ImageJ/Fiji) alone, several options are available. ThunderSTORM, for example, is despite its age (published in 2014, thereby lacking ways to correctly analyse sCMOS data) still widely used as it offers easy and robust access to 2D and especially 3D SMLM (astigmatism!). Unfortunately, ThunderSTORM is not actively maintained anymore. Another promising and more recent package is MARS (https://elifesciences.org/articles/75899), which focuses more on handling of single-molecule data (including, for example, smFRET) rather than providing extensive options to perform high accuracy and precision analysis of SMLM data. GDSC SMLM is therefore a very welcome addition as the inclusion of many tools such as fitting options, camera calibrations, tracking, simulations etc into a single package promises to empower SMLM data analysis.

 The manuscript is well written and provides a good overview on the available modalities. Further information is available via an excellent documentation site (https://gdsc-smlm.readthedocs.io/en/v1.0.2/index.html) and raw data is provided for users to test the software (https://doi.org/10.6084/m9.figshare.20795677). Overall, I fully approve the article to be further indexed on online academic databases.

 Additional comments

1. Personally, I am a bit confused when the word “image” refers to an image/movie consisting of many frames (e.g. the doc chapter 3 refers to: “Finding and fitting spots on the image” (especially if “image” is then combined temporal (!) “median filter” then moving along frames in a movie. Maybe the authors could screen the text in the manuscript and the documentation to clean up potential issues or at least provide a clear definition.
2. As it stands, GDSC SMLM seems to target the advanced user as the variety of options are somewhat overwhelming (and could be better introduced or exemplified). The authors opted to include shortcuts here, e.g., the module “SimpleFit” which is supposed to “just” fit some data. When I run SimpleFit, however, choosing an sCMOS as camera, the analysis failed as sCMOS requires a camera profile. Again, this is explained somewhere in the documentation, but an easy solution could be to default to the NA option in case no profile is available. (NA camera option is again easily mixed up with N.A. referring to numerical aperture).
3. The 3D data analysis parts (astigmatism) is currently marked as not being robust (“This option is experimental and should not be used for 3D analysis”). What is the current status? Are the authors planning to add additional 3D functionalities (e.g., biplane/DH,..)?
4. Assuming that users of ThunderSTORM form a large group of potential users, how does GDSC SMLM perform performance wise (e.g. time to analyse the provided data versus achievable recall/precision etc?).
5. Along the same line, a set of step-by-step tutorials (maybe even complemented by a written macro) for beginner and advanced level could help to draw and keep additional users to the software.
6. Fourier Ring correlation: 2D and/or 3D?
7. Do the authors plan to support/extend the software in the near future?

Are the conclusions about the tool and its performance adequately supported by the findings presented in the article?

Yes

Is the rationale for developing the new software tool clearly explained?

Yes

Is the description of the software tool technically sound?

Yes

Are sufficient details of the code, methods and analysis (if applicable) provided to allow replication of the software development and its use by others?

Yes

Is sufficient information provided to allow interpretation of the expected output datasets and any results generated using the tool?

Yes

Reviewer Expertise:

single-molecule biophysics, super-resolution imaging

I confirm that I have read this submission and believe that I have an appropriate level of expertise to confirm that it is of an acceptable scientific standard.

# Peer review: Reviewer response for version 1

**DOI:** 10.21956/wellcomeopenres.20314.r52565

**Published:** 2022-11-16

**Authors:** Ulrike Endesfelder

**Affiliations:** 1 Institut für Mikrobiologie und Biotechnologie, Rheinische Friedrich-Wilhelms-Universität Bonn, Bonn, Germany

The GDSC SMLM software package is a ready-to-use SMLM toolbox integrated into the widely used open-source ImageJ software. It offers simple functionality, many options and optimized processing. In addition to localization fitting, a key step for any SMLM imaging experiment, it provides several tools to assess or improve image quality (e.g., filtering, PSF shape estimation, or FRC image resolution), implementations of widely used advanced analysis tools (e.g., single particle tracking or clustering), and a modeling tool to simulate single molecule images.

 Importantly, the performance of the PeakFit localizer (highly important part of the software as PSF fitting is the first processing step done on any SMLM raw data) was evaluated in detail in an SMLM community competition (and ranked highly in that evaluation).

 In addition, the software is provided as open source code and is well documented by a manual.

 In summary, this is an extremely valuable tool in the field of SMLM imaging. The software is fast and easy to use and has no technical shortcomings. Since it is implemented in ImageJ, it can be easily combined with other tools in the field, many of which are deployed in an ImageJ environment. Therefore, I strongly believe that the manuscript can be indexed as is. If the authors wish to revise it, there are some options that would help beginners in this field:

 The main part explained in the text is the PeakFit localizer. It would be nice to add each a short section on all the tools that come with the full package, even if they are tools developed by others and "just" integrated into the package. Most tools are referenced citing the original paper, but there is no explanation of how they work.

 Similarly, pseudocode would help beginners understand how each tool works. In addition, a visualization of the processing steps under the given settings on demo use cases of the different tools would also be great. The authors provide demo data, but do not document their use with the software (except fitting with PeakFit).

Are the conclusions about the tool and its performance adequately supported by the findings presented in the article?

Yes

Is the rationale for developing the new software tool clearly explained?

Yes

Is the description of the software tool technically sound?

Yes

Are sufficient details of the code, methods and analysis (if applicable) provided to allow replication of the software development and its use by others?

Yes

Is sufficient information provided to allow interpretation of the expected output datasets and any results generated using the tool?

Yes

Reviewer Expertise:

Single-molecule microscopy and tracking, microbiology

I confirm that I have read this submission and believe that I have an appropriate level of expertise to confirm that it is of an acceptable scientific standard.
